# Serum angiopoietin-1 and angiopoietin-2 levels in Turkish patients with Behçet’s disease: associations with clinical manifestations and inflammatory markers

**DOI:** 10.55730/1300-0144.6200

**Published:** 2026-02-09

**Authors:** Sercan GÜCENMEZ, Hakan EMMUNGİL, Figen YARGUCU ZİHNİ, Gökhan KESER, Kenan AKSU

**Affiliations:** Division of Rheumatology, Department of Internal Medicine, Faculty of Medicine, Ege University, İzmir, Turkiye

**Keywords:** Angiogenesis, Behçet’s disease, vasculitis

## Abstract

**Background/aim:**

Emerging evidence suggests that dysregulated angiogenesis may contribute to the pathogenesis of Behçet’s disease (BD). This study primarily aimed to investigate serum angiopoietin-1 (Ang-1) and angiopoietin-2 (Ang-2) levels in Turkish patients with BD and compare them with healthy controls. The secondary aims were to compare angiopoietin levels based on clinical manifestations, disease activity, and medication use and to investigate their correlations with age, disease duration, and inflammatory markers.

**Materials and methods:**

This cross-sectional study included 56 consecutive patients with BD, diagnosed according to International Study Group criteria, and 56 age- and sex-matched healthy controls. Physical characteristics, clinical manifestations, time since symptom onset, time since diagnosis, medications, erythrocyte sedimentation rate (ESR), and C-reactive protein (CRP) levels were recorded. Serum Ang-1 and Ang-2 levels were measured by enzyme-linked immunosorbent assay. Comparisons between the two groups were analyzed using the Student t-test or Mann–Whitney U test, and correlations were assessed using Pearson or Spearman correlation coefficients, depending on the data distribution.

**Results:**

Serum Ang-1 and Ang-2 levels were significantly higher in patients with BD compared to healthy controls (p < 0.001 and p = 0.031, respectively). Patients with genital ulcers had significantly lower serum Ang-1 levels compared to those without (p = 0.029). Additionally, Ang-2 levels showed moderate positive correlations with CRP (r = 0.518, p < 0.001) and ESR (r = 0.459, p < 0.001).

**Conclusion:**

The elevated expression of Ang-1 and Ang-2 in Turkish patients with BD supports the growing evidence of their possible roles in BD pathogenesis.

## 1. Introduction

Behçet’s disease (BD) is a multisystem vascular disorder of unknown etiology, generally characterized by recurrent episodes of oral aphthous ulcers, genital ulcers, cutaneous lesions, and ocular involvement [1]. While the mucocutaneous and ocular manifestations are hallmark characteristics of BD, other systemic symptoms including musculoskeletal, central nervous system, and gastrointestinal involvement may accompany the clinical picture [2,3]. BD exhibits a unique geographical distribution, with a higher prevalence in the Mediterranean, Middle Eastern, and East Asian regions. Among these regions, Türkiye is one of the countries with the highest reported prevalence, with BD cases estimated to range from 20 to 421 per 100,000 adults [4–6].

The pathogenesis of BD remains only partially understood; however, a complex interplay between genetic predisposition and environmental or infectious triggers contributing to immune dysregulation is the most widely accepted theory [7]. Both innate and adaptive immunity are involved in disease progression, with key roles played by T lymphocytes and neutrophils, ultimately leading to tissue damage [8,9]. Imaging studies have demonstrated endothelial dysfunction and vascular abnormalities in BD, supporting the major role of endothelial activation in the pathology of the disease [10]. In line with this, emerging evidence suggests that dysregulated angiogenesis may contribute to BD pathogenesis [11,12]. Increased levels of angiogenic factors, such as vascular endothelial growth factor and monocyte chemoattractant protein-1 [13], as well as the overexpression of antiangiogenic factors like angiostatin, have been reported in patients with BD [14].

Angiopoietins serve as key regulators of vascular integrity and endothelial stability. Angiopoietin-1 (Ang-1) and angiopoietin-2 (Ang-2) are antagonistic ligands that bind to the extracellular domain of the Tie-2 receptor, predominantly expressed by endothelial cells [15]. Their balance plays a crucial role in vascular homeostasis [16]. While Ang-1 binding to Tie-2 promotes endothelial cell survival and migration through rapid receptor auto-phosphorylation, Ang-2 antagonizes Tie-2 activation, disrupting endothelial integrity and enhancing vascular permeability [16,17].

Previous studies have reported conflicting results regarding angiopoietin levels in BD. Choe et al. [18] found elevated Ang-1 expression in Korean patients with BD compared to healthy controls, whereas Bassyouni et al. [19] reported significantly lower Ang-1 levels in Egyptian patients with BD, particularly in those with vascular involvement. However, despite these discrepancies, the findings consistently highlight the significance of angiopoietin dysregulation in the pathogenesis of BD [12]. To better understand the role of Ang-1 and Ang-2 in BD, this study was conducted in Türkiye, where BD is reported with a relatively higher prevalence. The primary aim was to investigate serum Ang-1 and Ang-2 levels in Turkish patients with BD and healthy controls. The secondary aims were to compare angiopoietin levels based on clinical manifestations, disease activity, and medication use and to investigate their possible associations with age, disease duration, and inflammatory markers.

## 2. Materials and methods

This cross-sectional study was conducted in the Rheumatology Clinic of the Ege University Faculty of Medicine Hospital. The study was approved by the institutional ethics committee (date: 30 May 2012; approval number: 12-5.1/7). Written informed consent was obtained from all participants prior to study enrollment. Patients were consecutively recruited between June 2012 and June 2013 from the outpatient rheumatology clinic of the same institution.

### 2.1. Participants

Patients aged 18 years or older with BD, diagnosed according to International Study Group criteria [20], were included in the study. Exclusion criteria included the presence of malignancy, acute or chronic infections, pregnancy, or lactation. To minimize clinical and laboratory heterogeneity, patients with coexisting systemic autoimmune diseases such as rheumatoid arthritis or systemic lupus erythematosus were also excluded. The control group consisted of age- and sex-matched healthy volunteers, who were either relatives of the enrolled BD patients or individuals accompanying other patients at the outpatient clinic.

### 2.2. Outcome measures

#### 2.2.1. Physical and disease-related characteristics

Physical characteristics (age and sex) and smoking status were recorded for both groups. Time since symptom onset, time since diagnosis, and current medications were obtained from patient records for the patients with BD. The use of azathioprine, corticosteroids, and cyclophosphamide was recorded separately as binary variables (present/absent), regardless of whether they were used alone or in combination. Erythrocyte sedimentation rate (ESR) and C-reactive protein (CRP) were measured from venous blood samples collected from the forearm during regular visits.

#### 2.2.2. Clinical manifestations

The clinical manifestations of BD were assessed and recorded as present or absent based on combined information obtained from the patient’s medical history and a physical examination, including mucocutaneous, musculoskeletal, vascular, gastrointestinal, ocular, and central nervous system involvement. For each patient, BD-related features such as oral and genital ulcers, papulopustular (acneiform-like) and nodular (erythema nodosum-like) skin lesions, uveitis, arthritis, and arterial or venous thrombosis were documented. Parenchymal and extraparenchymal neurological findings, as well as ulcerative or aphthous gastrointestinal lesions, were recorded only for patients who reported relevant symptoms, and further investigations (e.g., imaging or endoscopy) were performed by relevant departments (neurology, gastroenterology, etc.) when clinically indicated. Given the multisystemic nature of BD, individual patients often exhibited multiple concurrent manifestations; therefore, each manifestation was recorded as a separate binary variable (present/absent), regardless of cooccurrence.

Disease activity was defined as the presence of at least one active BD-related clinical manifestation at the time of assessment, including mucocutaneous and/or systemic involvement, based on a clinical examination supported by contemporaneous clinical information. However, mild or isolated findings, such as oral ulcers in the absence of other systemic involvement, were not classified as active disease unless judged to be clinically significant by the assessing physician. This pragmatic approach was used to avoid overestimating disease activity in patients with minimal or stable symptoms.

#### 2.2.3. Serum angiopoietin-1 and angiopoietin-2 levels

Venous blood samples were collected from all participants and serum was separated by centrifugation. The obtained serum samples were stored at −80 °C until analysis. Serum Ang-1 and Ang-2 levels were measured by enzyme-linked immunosorbent assay (ELISA) for both the patients with BD and the healthy controls using commercially available kits (R&D Systems Inc., Minneapolis, MN, USA), following the manufacturer’s recommendations. Briefly, assay diluent and serum samples were added to antibody-coated microplate wells and incubated at room temperature. Following incubation, the wells were washed and a conjugated detection antibody was added. After further incubation and washing steps, the substrate solution was applied and the enzymatic reaction was stopped using a stop solution. Absorbance was measured at 450 nm using a microplate reader (ELx800, BioTek, Winooski, VT, USA). Concentrations of Ang-1 and Ang-2 were calculated based on the standard curves generated for each assay.

### 2.3. Statistical analysis

Statistical analysis was performed using IBM SPSS Statistics 30.0 (IBM Corp., Armonk, NY, USA). The Shapiro–Wilk test was used to assess the normality of continuous variables. Numerical data were presented as mean ± standard deviation for homogeneously distributed variables and as median (minimum/maximum) for heterogeneously distributed variables. Categorical data were expressed as frequency (n) and percentage (%). The statistical significance of comparisons between the two groups was determined using either the Student t-test for homogeneous data or the Mann–Whitney U test for heterogeneous data. Depending on the parametric assumptions, the Pearson correlation coefficient (r) or Spearman rank correlation coefficient (rho) was used for analysis, with r and rho values interpreted as follows: 0.10–0.39 = weak, 0.40–0.69 = moderate, 0.70–0.89 = strong, and 0.90–1.00 = very strong [21].

## 3. Results

This study was completed with 56 patients with BD (mean age: 40.9 ± 10.3 years) and 56 age- and sex-matched healthy controls (mean age: 41.5 ± 9.8 years). Physical characteristics and disease-related data are presented in Table 1. The mean serum Ang-1 and Ang-2 concentrations were significantly higher in patients with BD compared to healthy controls (p < 0.001 and p = 0.031, respectively).

In addition to oral ulcers, which were reported by all patients, the most common previously documented clinical manifestations were genital ulcers (78.6%), acneiform lesions (53.6%), ocular involvement (46.4%), and vascular involvement (44.6%). Among the patients with BD, vascular involvement was documented in 27 cases. Of these, 22 patients exhibited venous manifestations, including 9 cases involving large veins and 13 with deep vein thrombosis. Arterial involvement was present in 5 patients. Ocular involvement, specifically uveitis, was observed in 26 patients. Neuro-BD was reported by 5 patients. Among the 13 patients classified as having active disease at the time of the assessment, active systemic involvement was most frequently vascular (n = 8), followed by ocular involvement (n = 3), with fewer cases of mucocutaneous (n = 1) and neurological (n = 1) activity.

Patients with genital ulcers exhibited significantly lower serum Ang-1 levels compared to those without (p = 0.029). However, no significant differences were observed in median serum Ang-1 and Ang-2 levels concerning other specific clinical manifestations, disease activity status, or medication use (p > 0.05) (Table 2).

While no significant correlations were found between serum Ang-1 levels and age, time since symptom onset, time since diagnosis, or laboratory parameters (p > 0.05), serum Ang-2 levels demonstrated moderate correlations with CRP (r = 0.518, p < 0.001) and ESR (r = 0.459, p < 0.001) (Table 3).

## 4. Discussion

This study aimed to investigate serum Ang-1 and Ang-2 levels in patients with BD and healthy controls; compare Ang-1 and Ang-2 levels based on clinical manifestations, disease activity, and medication use; and explore their possible correlations with age, time since symptom onset, time since diagnosis, and inflammatory markers. Our findings revealed that both Ang-1 and Ang-2 levels were significantly higher in patients with BD compared to healthy controls, suggesting a potential role of angiopoietins in BD-related vascular pathology. Furthermore, Ang-1 levels were found to be lower in the presence of genital ulcers. While this observation raises the possibility of a modulatory or protective role for Ang-1, the cross-sectional nature of the study precludes causal interpretation. In contrast, Ang-2 levels were correlated with CRP and ESR as systemic inflammation markers. The upregulation of Ang-1 and Ang-2 levels in patients with BD may reflect an attempt to counteract endothelial instability and chronic inflammation. While increased Ang-1 levels could serve as a compensatory mechanism to maintain vascular integrity, elevated Ang-2 may contribute to persistent endothelial activation and increased vascular permeability [22]. However, the cross-sectional nature of the study limits our ability to determine whether these elevations are disease-specific or a reflection of systemic inflammation.

While the role of angiopoietins has been extensively explored in other inflammatory disorders, studies on BD are limited. To the best of our knowledge, only two studies in the literature have investigated the role of angiopoietins in BD. Choe et al. [18] conducted a study involving 59 patients with BD and 65 healthy controls and found significantly higher serum Ang-1 levels in patients with BD compared to the control group, which aligns with our results. In their study [18], although serum Ang-2 levels were also observed to be higher in patients with BD compared to the control group, this difference did not reach statistical significance. On the other hand, Bassyouni et al. [19] conducted a study involving 47 patients with BD and 30 healthy controls to investigate plasma Ang-1 levels and, unlike our findings and those of Choe et al., reported significantly lower Ang-1 levels in patients with BD compared to the control group. The discrepancies in angiopoietin levels observed across studies may be attributed to multiple factors, including differences in clinical presentation, disease activity status, and methodological variations such as using serum versus plasma. Notably, disease activity may also play a role; however, comparisons are limited by the fact that these three studies used different criteria to define active disease and none employed a standardized disease activity index. Although disease activity in our study was determined through expert physician assessment based on clinical examination rather than a formal scoring system, our findings are consistent with the literature in suggesting that serum angiopoietin levels do not appear to reflect the clinical activity of BD.

Beyond the comparison of overall angiopoietin levels between patients with BD and healthy controls, we further explored whether specific clinical manifestations influenced Ang-1 and Ang-2 levels. In our study, only patients with genital ulcers exhibited significantly lower Ang-1 levels, while no significant differences were observed for other clinical features. Interestingly, Bassyouni et al. [19] reported lower Ang-1 levels in patients with vascular involvement, whereas Choe et al. [18] found higher Ang-1 levels in those with skin involvement. However, the distribution of clinical subgroups varied substantially across studies. In the cohort of Choe et al. [18], the prevalence of vascular and genital involvement was relatively low at 3.4% and 13.6%, respectively, while in the study conducted by Bassyouni et al. [19], vascular involvement was reported in 19.1% of patients. In contrast, our study included a much higher proportion of patients with genital ulcers (78.6%), while vascular involvement was observed in 44.6% of patients. These differences suggest that the absence or presence of statistical significance in angiopoietin levels across specific clinical subgroups may be influenced by the size and distribution of these subgroups. Therefore, subgroup analyses should be interpreted with caution, particularly when evaluating vascular involvement, which plays a central role in the pathophysiology of BD. The limited number of patients in some subgroups may have affected the statistical power to detect true differences or may have increased the likelihood of chance findings. Moreover, all three studies, including ours, had a cross-sectional design, which inherently reflects a specific time frame and patient distribution. This may partially explain the variation in subgroup-related findings across studies, as the observed clinical manifestations are likely influenced by the characteristics of the patient population encountered during that specific study period. Although the direction and pattern of change differ across studies, the recurrent observation of altered Ang-1 levels in relation to various clinical manifestations suggests a possible involvement of Ang-1 in disease expression. However, whether these changes reflect a protective mechanism, a response to ongoing inflammation, or an epiphenomenon remains unclear and warrants further investigation.

Additionally, given the number of comparisons performed across different clinical features, the possibility of both type I and type II errors should be considered. These analyses were exploratory in nature due to the limited number of patients in certain subgroups and should therefore be interpreted with caution. The association between Ang-1 levels and genital ulcers should be interpreted as hypothesis-generating and confirmed in future studies.

Beyond clinical manifestations, treatment-related factors were also considered, as immunosuppressive medications could potentially influence angiopoietin levels. According to the current recommendations of the European Alliance of Associations for Rheumatology for the management of BD, immunosuppressive and immunomodulatory therapies are frequently required to control mucocutaneous, ocular, vascular, and other systemic manifestations [23]. Consequently, many patients were receiving ongoing treatment at the time of clinical and laboratory assessment. Although treatment-naive populations may be desirable for biomarker research, the multisystemic and relapsing nature of BD makes this difficult in routine clinical practice. Therefore, the potential influence of treatment should be considered when interpreting angiopoietin levels in this patient population. Bassyouni et al. [19] reported significantly higher Ang-1 levels in patients receiving cyclophosphamide or corticosteroids compared to those not receiving these medications. However, neither our study nor that of Choe et al. [18] found significant differences in Ang-1 or Ang-2 levels based on the presence or absence of specific immunosuppressive therapies. While our results suggest that medication use did not significantly affect angiopoietin levels in our cohort, we acknowledge that the complex treatment combinations and limited subgroup sizes warrant cautious interpretation of the results.

In our study, serum Ang-1 levels showed no significant correlations with age, time since symptom onset, time since diagnosis, ESR, or CRP levels. However, serum Ang-2 levels demonstrated moderate positive correlations with both CRP (r = 0.518, p < 0.001) and ESR (r = 0.459, p < 0.001). Similarly, Choe et al. [18] reported a significant correlation between Ang-2 levels and ESR (r = 0.306, p = 0.018), but no association with CRP. Their study also identified a positive correlation between disease duration and Ang-1 levels (r = 0.320, p = 0.015), although Ang-1 did not reflect clinical or laboratory disease activity markers. On the other hand, Bassyouni et al. [19] did not find any significant correlations between plasma Ang-1 levels and age, disease duration, or inflammatory markers.

Conducting this study in Türkiye, where BD is more prevalent, is a strength of the study. On the other hand, this study has several limitations. First, its cross-sectional design does not allow for the assessment of causality or the evaluation of changes in angiopoietin levels over time. Furthermore, the cross-sectional design and the lack of systematically recorded longitudinal data such as relapse history or thrombotic events limited the evaluation of clinical outcomes that could have enhanced the interpretability of angiopoietin levels as biomarkers. Second, no validated disease activity index was used, which limited our ability to interpret the relationship between disease activity and angiopoietin levels. Additionally, further diagnostic evaluations were performed only for patients who reported symptoms related to a specific system. This approach may have limited the detection of asymptomatic or subclinical involvement, potentially restricting a more comprehensive assessment of disease involvement. Finally, although we analyzed the associations between angiopoietins and clinical features, the sample sizes of certain subgroups may have limited the statistical power, limiting the robustness of our results.

In conclusion, our study demonstrated that serum Ang-1 and Ang-2 levels were significantly elevated in patients with BD compared to healthy controls, suggesting a potential role for these molecules in BD-associated vascular pathology. Additionally, the association between lower Ang-1 levels and the presence of genital ulcers raises the possibility that Ang-1 may be involved in disease expression, although the direction and nature of this relationship remain unclear. The moderate correlations between Ang-2 and systemic inflammation markers may be of interest for future research. Future longitudinal and/or prospective studies with larger sample sizes and standardized methodologies are needed to establish causality between angiopoietin levels and clinical manifestations in BD, and mechanistic studies may help further clarify whether these associations reflect secondary effects.

## Figures and Tables

**Table 1:** Physical and disease-related characteristics.

	Behçet’s disease (n=56)	Healthy controls (n=56)	p
**Age (years)**	40.9 ± 10.3	41.5 ± 9.8	0.764^*^
**Sex, male (n, %)**	35 (62.5)	35 (62.5)	1.000**
**Current smoking (Yes, n, %)**	13 (23.2)	22 (39.3)	0.119**
**Time since diagnosis (months)**	31.45 ± 9.5	NA	NA
**Time since symptom onset (years)**	8 (3/30)	NA	NA
**Laboratory investigations**			
C- reactive protein (mg/L)	0.46 (0.03/22.09)	NA	NA
Erythrocyte sedimentation rate (mm/h)	9.5 (2.0/96.0)	NA	NA
Angiopoietin-1 (ng/mL)	45.44 ± 12.14	36.90 ± 8.31	**<0.001** ^*^
	8.54 (4.64–12.44)†		
Angiopoietin-2 (pg/mL)	2102.9 ± 655.1	1879.3 ± 395.7	**0.031** ^*^
	223.59 (20.42–426.76)†		
**Current medications (n, %)**			
Azathioprine	32 (57.1)	NA	NA
Corticosteroids	22 (39.3)	NA	NA
Cyclophosphamide	4 (7.1)	NA	NA

NA: Not applicable. Values given in bold font: p < 0.05.

* Student t-test;

** Pearson chi-square test;

† mean difference (95% confidence interval)

**Table 2:** Comparison of serum angiopoietin-1 and angiopoietin-2 levels based on clinical manifestations in Behçet’s disease.

n= 56	n (%)	Angiopoietin-1 (ng/mL)			Angiopoietin-2 (pg/mL)		
		Present median (min/max)	Absent median (min/max)	p*	Present median (min/max)	Absent median (min/max)	p*
**Genital ulcers**	44 (78.6)	41.22 (23.30/74.47)	55.49 (24.97/68.55)	0.029	2150.4 (1153.9/3662.9)	1946.4 (1137.8/2972.8)	0.385
**Acneiform lesions**	30 (53.6)	43.24 (23.30/74.47)	43.15 (24.97/64.40)	0.980	2129.9 (1137.8/3662.9)	1946.4 (1177.0/3532.4)	0.565
**Erythema nodosum like lesions**	22 (39.3)	47.97 (25.75/69.10)	41.76 (23.30/74.47)	0.287	1946.4 (1137.8/3662.9)	2105.1 (1177.0/3161.8)	0.718
**Ocular involvement**	26 (46.4)	43.82 (23.30/68.55)	41.53 (24.97/74.47)	0.495	2065.4 (1177.0/3021.7)	2030.7 (1137.8/3662.9)	0.818
**Articular involvement**	12 (21.4)	46.12 (31.41/62.57)	42.56 (23.30/74.47)	0.905	1837.5 (1304.7/3662.6)	2105.1 (1137.8/3532.4)	0.590
**Vascular involvement**	25 (44.6)	49.55 (24.97/74.47)	40.63 (23.30/68.55)	0.115	2333.6 (1137.8/3532.4)	1877.1 (1177.0/3662.9)	0.204
**Neuro-Behçet’s**	5 (8.9)	43.06 (29.62/52.90)	43.42 (23.30/74.47)	0.596	1906.8 (1226.1/3161.8)	2115.0 (1137.8/3662.9)	0.557
**Current medications use**	37 (66.1)	44.23 (25.75/74.48)	42.07 (23.31/64.40)	0.659	1906.8 (1137.9/3532.42)	2463.0 (1177.0/3662.85)	0.126
Azathioprine	32 (57.1)	44.41 (25.75/74.48)	42.57 (23.31/64.40)	0.529	1946.43 (1137.8/3532.42)	2139.8 (1177.0/3662.85)	0.585
Corticosteroids	22 (39.3)	46.48 (25.75/74.48)	41.53 (23.31/68.55)	0.261	1891.94 (1137.8/2972.83)	2174.6 (1177.0/3662.85)	0.144
Cyclophosphamide	4 (7.1)	39.43 (31.41/48.92)	43.46 (23.31/74.48)	0.362	1901.84 (1255.6/2095.18)	2139.85 (1137.8/3662.85)	0.362
**Active Disease**	13 (23.2)	52.90 (29.62/69.10)	41.44 (23.30/74.47)	0.229	2095.2 (1153.39/3532.4)	2035.7 (1137.8/3662.9)	0.363

* Mann–Whitney U test.

**Table 3:** Correlation between angiopoietins and clinical and demographic variables in Behçet’s disease.

n=56		Angiopoietin-1	Angiopoietin-2
Age (years)	r	0.102	0.173
	p	0.456	0.203
Time since symptom onset (years)	rho	0.027	0.219
	p	0.846	0.105
Time since diagnosis (months)	r	−0.097	0.070
	p	0.479	0.609
C- reactive protein (mg/L)	rho	−0.021	0.518
	p	0.885	**<0.001**
Erythrocyte sedimentation rate (mm/h)	rho	0.004	0.459
	p	0.979	**<0.001**

r: Pearson correlation coefficient, rho: Spearman correlation coefficient.

Values given in bold font: r or rho ≥ 0.4 and p < 0.05.
